# Low iron mitigates viral survival: insights from evolution, genetics, and pandemics—a review of current hypothesis

**DOI:** 10.1186/s43042-020-00114-z

**Published:** 2020-12-16

**Authors:** Rahma Menshawey, Esraa Menshawey, Ayman H. K. Alserr, Antoine Fakhry Abdelmassih

**Affiliations:** 1 Faculty of Medicine, Kasr al Ainy, Cairo University, Geziret Elroda, Manial, Cairo, 11562 Egypt; 2 Vascular Surgery Department, Cairo University Hospitals, Giza, Egypt; 3 Pediatric Cardiology Department, Cairo University Hospitals, Giza, Egypt

**Keywords:** Iron deficiency anemia, RNA viruses, COVID-19, Hyperferritinemia, Hepcidin, Ferroptosis

## Abstract

**Background:**

Upon re-examination of our human history, evolutionary perspectives, and genetics, a prevailing iron deficiency phenotype appears to have evolved to protect the human race from extinction.

**Body:**

In this review, we summarize the evolutionary and genetic perspectives pointing towards the hypothesis that low iron mitigates infection. The presence of infection promotes the generation of resistance alleles, and there are some evolutionary and genetic clues that suggest the presence of an iron deficiency phenotype that may have developed to protect against infection. Examples include the relative paucity of iron overload genes given the essential role of iron, as well as the persistence of iron deficiency among populations in spite of public health efforts to treat it. Additional examination of geographic areas with severe iron deficiency in the setting of pandemics including H1N1, SARS, and COVID-19 reveals that areas with higher prevalence of iron deficiency are less affected. RNA viruses have several evolutionary adaptations which suggest their absolute need for iron, and this dependency may be exploited during treatment.

**Conclusion:**

RNA viruses pose a unique challenge to modern healthcare, with an average of 2–3 new pathogens being discovered yearly. Their overarching requirements for iron, along with human evolutionary and genetic adaptations which favored an iron deficiency phenotype, ultimately suggest the potential need for iron control in these infections.

## Background

Ancient stars in their death throes spat out atoms like iron which this universe had never known. ... Now the iron of old nova coughing's vivifies the redness of our blood. Howard Bloom

Coronavirus Disease 2019 (COVID-19) is a viral infection caused by the newly discovered Severe Acute Respiratory Syndrome Coronavirus 2 (SARS-Cov-2). It was first discovered in the city of Wuhan, China, in December 2019, and by March 11, 2020, the World Health Organization (WHO) declared it as a global health emergency [1]. Recent findings and lab data suggests that COVID-19 falls within the spectrum of hyperferritinemia syndromes given the shared manifestations of macrophage activation syndrome (MAS), multiple organ dysfunction (MOD), and septic shock [2]. The nature of this ribonucleic acid (RNA) virus, along with findings of hyperferritinemia [3], may be responsible for the resulting increase in intracellular iron, which is the main trigger for ferroptosis within cells. These findings suggest an underlying role of iron in the pathogenesis of COVID-19.

Advancements in our civilization may have increased our risk to infections and disease, as well as stressed our survival fitness. Overcrowding, domestication of animals, and the agrarian revolution may have all played a role in the development of a prevailing protective iron deficiency phenotype, which may have increased our resistance as a species to acute infections and epidemics [4]. In this review, using insights from evolution and genetics, we summarize the hypothesis that low iron may mitigate infection, as well as the potential benefits of iron control in the setting of infections, such as the current pandemic.

## Main text

### 1. Iron, iron deficiency, anemia, and infection

#### i. Iron’s role in infection

Iron is a vital element required in the internal processes and cellular operations of nearly all multi-cellular organisms [5]. Uniquely, bacteria, fungi, and some viruses have developed methods to extract iron from their hosts [6]. Some viruses infect iron-acquiring cells by binding to transferrin receptor type 1, while other viruses target the Human Homeostatic Iron regulator Protein (HFE) genes and hepcidin, with the end goal of inducing iron overload on a cellular level to promote *their* survival and replication [7]. For example, the cytomegalovirus (CMV) interferes with the Major Histocompatibility Complex (MHC) class I proteins causing the proteosomal degradation of HFE, reversing the hepcidin effect of reducing cellular iron uptake, allowing cells which they infect to become overloaded with iron [8].

In a study regarding the correlation between iron levels and viral load, Chang et al. found that there was reduced virulence within iron deficient cells [9]. It was further demonstrated that iron-chelation benefits the host by reducing the amount of viral nucleic acids and proteins, which in turn decreases viral replication and release. Additionally, low iron levels could downregulate the expression of adhesion molecules required for viral attachment and internalization [9–11]. Notably, iron intake that exceeds the body's needs may promote the proliferation of pathogens, while some evidence suggests that a state of hypoferremia can be protective in endemic areas. Dietary iron restriction may protect against infection in settings of high transmission or morbidity [12]. One study suggests that prophylactic daily supplementation of iron and folic acid, with or without zinc, showed no significant differences in attack rates of respiratory infection, dysentery, or diarrhea. Interestingly, this study had originally hypothesized that iron supplementation would mitigate mortality risk in their cohort of Nepali children with iron deficiency anemia, but the rates of mortality and morbidity did not differ between treatment and placebo groups [13]. This study suggests that the presence of concurrent malnutrition (i.e., protein energy malnutrition) disorders limit the effectiveness of iron therapy in the setting of iron deficiency anemia (IDA), while other studies suggest that the resistance to iron supplementation in the setting of anemia may be due to genetic variations. When controlling for the presence of chronic illness and malabsorption diseases in patients with IDA, analysis revealed that the presence of genetic variants (T495M and P555S) is responsible for non-response to parenteral iron therapy [14, 15]. Contrastingly, cell-mediated immunity is affected by iron deficiency, as demonstrated by Das et al. who found that patients with IDA had significantly lower levels of CD4+ T cells (*P* < 0.05), as well as a declining CD4 to CD8 cell ratio. Cell-mediated immunity (CMI) was improved with iron supplementation for 3 months [16]. The goal remains in finding the *optimal iron status in the setting of infection *[17] and finding the trade off point between its effects on immunity in the setting of infection. As suggested by Wander et al., a compromise will exist between the effects of hypoferremia on CMI, and the need for resisting certain infections, and local disease ecology [12].

#### ii. Iron deficiency anemia, females, and COVID-19 infection

IDA is a type of anemia defined as inadequate tissue oxygenation caused by abnormal red blood cells as a result of a defective iron state, which is prevalent worldwide. Females are more commonly affected owing to the chronic blood loss during menstruation and pregnancy [18]. According to the WHO, global anemia prevalence is 30.2% (95% CI 28.7–31.6)/468 million (95% CI 446–491) among non-pregnant women of reproductive age (WRA). Of the number of individuals affected by anemia worldwide, 29% were non-pregnant WRA, while 16% of those affected were males. The highest prevalence was found in Africa (47.5%) and South East Asia (45.7%), while the lowest prevalence was in the Americas (17.8%) [19, 20]. Studies suggest that many intracellular microorganisms, such as plasmodia and mycobacteria, are enhanced by iron therapy; while there appears to be a decreased susceptibility to malaria-related illness [21, 22], human immunodeficiency virus (HIV) [23, 24], and tuberculosis (TB) [25, 26] in patients with IDA. Oral iron supplementation has been associated with increased risk of infection and morbidity. In fact, treatment of anemia in malaria endemic areas is not recommended without prophylaxis for malaria [24, 27]. The right balance must be found between dose and timing of intervention in areas with endemic infectious disease [28]. Given that IDA has higher prevalence among females worldwide, this deficiency may have a potential protective factor for females in the setting of COVID-19 infection.

COVID-19 patient data suggests a decreased risk of infection and decreased risk of poor outcome (either intensive care unit (ICU) admission or mortality) in females [29–31]. Sharma et al. outlined the differences in mortality between sexes during the COVID-19 pandemic, and concluded that males were more likely to develop severe disease, and had higher mortality and case fatality ratio compared to females [32]. Reports from Italy have shown higher death rates for males compared to females across all age groups, with a male to female death ratio of 80 to 20% [33]. Moreover, SARS-COV, as well as Middle Eastern Respiratory Syndrome (MERS)-COV, also affected more males than females [34]. Mortality appears to be related to the presence of risk factors including cardiovascular disease, which have higher occurrence in men. It is posited that females are more protected than males owing to the role of estrogen and the X chromosome on the immune system, as well as social behavioral differences between males and females that favor female overall health, such as willingness to seek preventative care [32, 35].

In China, an interesting observation was made, in that in pregnant females, no increased risk for COVID-19 was observed. In another study, 92% presented with mild disease and only 8% (9 patients) had severe hypoxemia. Moreover, of those 9 patients with severe disease, 6 of them developed this only after delivery [36]. This observation warrants more robust research. Reduced iron availability in pregnant females may be protective. The prevalence of anemia in pregnant females may range from anywhere between 35–75% in developing countries and 18% in developed countries [37, 38]. During fetal development, iron being shunted from the mother to the fetus may result in IDA, which may be a protective factor for pregnant females in the setting of COVID-19. The placenta itself retains approximately 90 mg of iron for its own functions, as well as shunts 270 mg of iron to the fetus, throughout the second and third trimesters [39, 40].

**Fig. 1:**
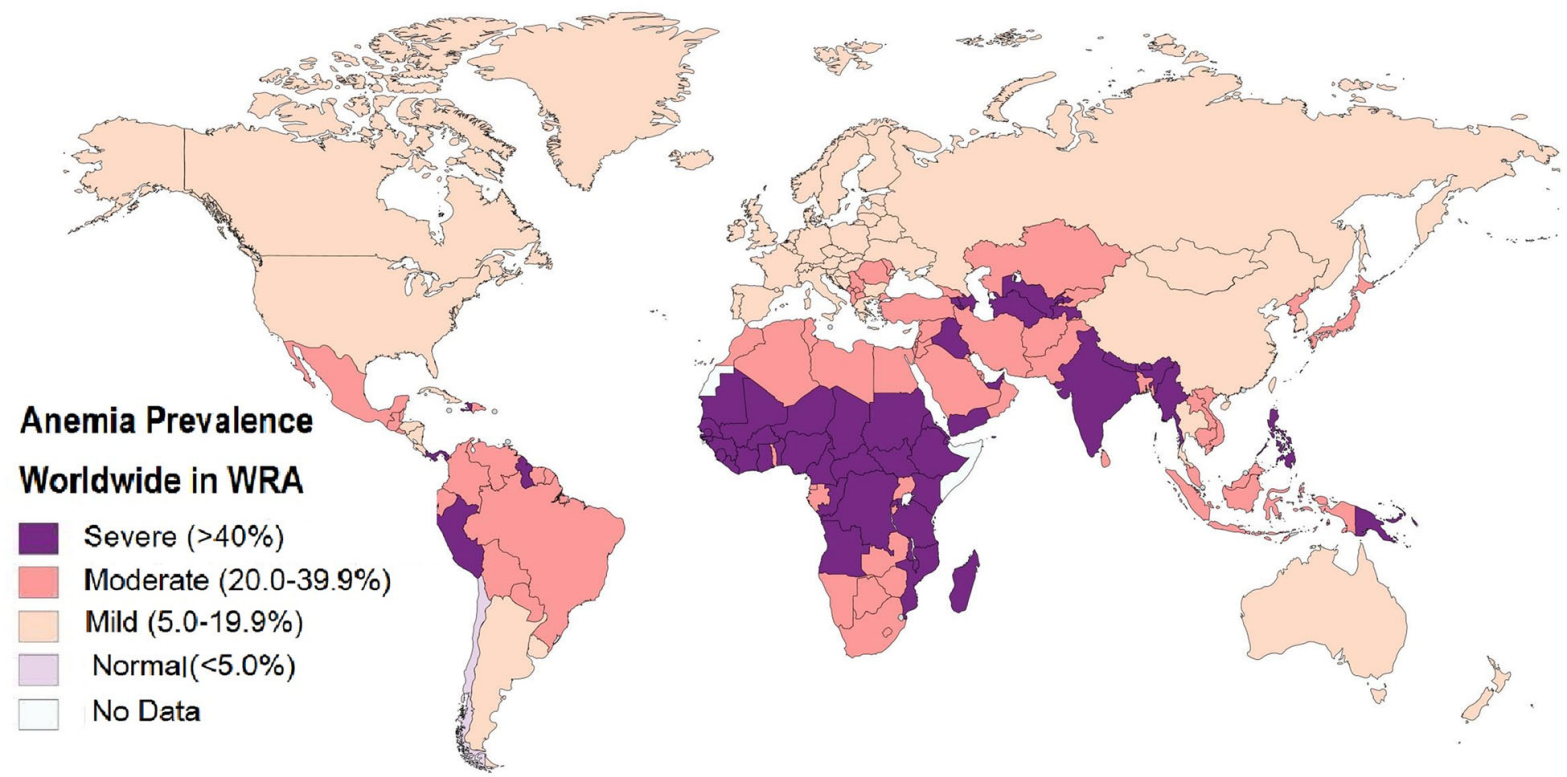
Anemia prevalence worldwide in women of reproductive age. The highest prevalence is seen in Sub-Saharan Africa (SSA), Eastern Mediterranean, and South East Asia. The lowest prevalence is in the Americas. Data source: WHO

**Fig. 2:**
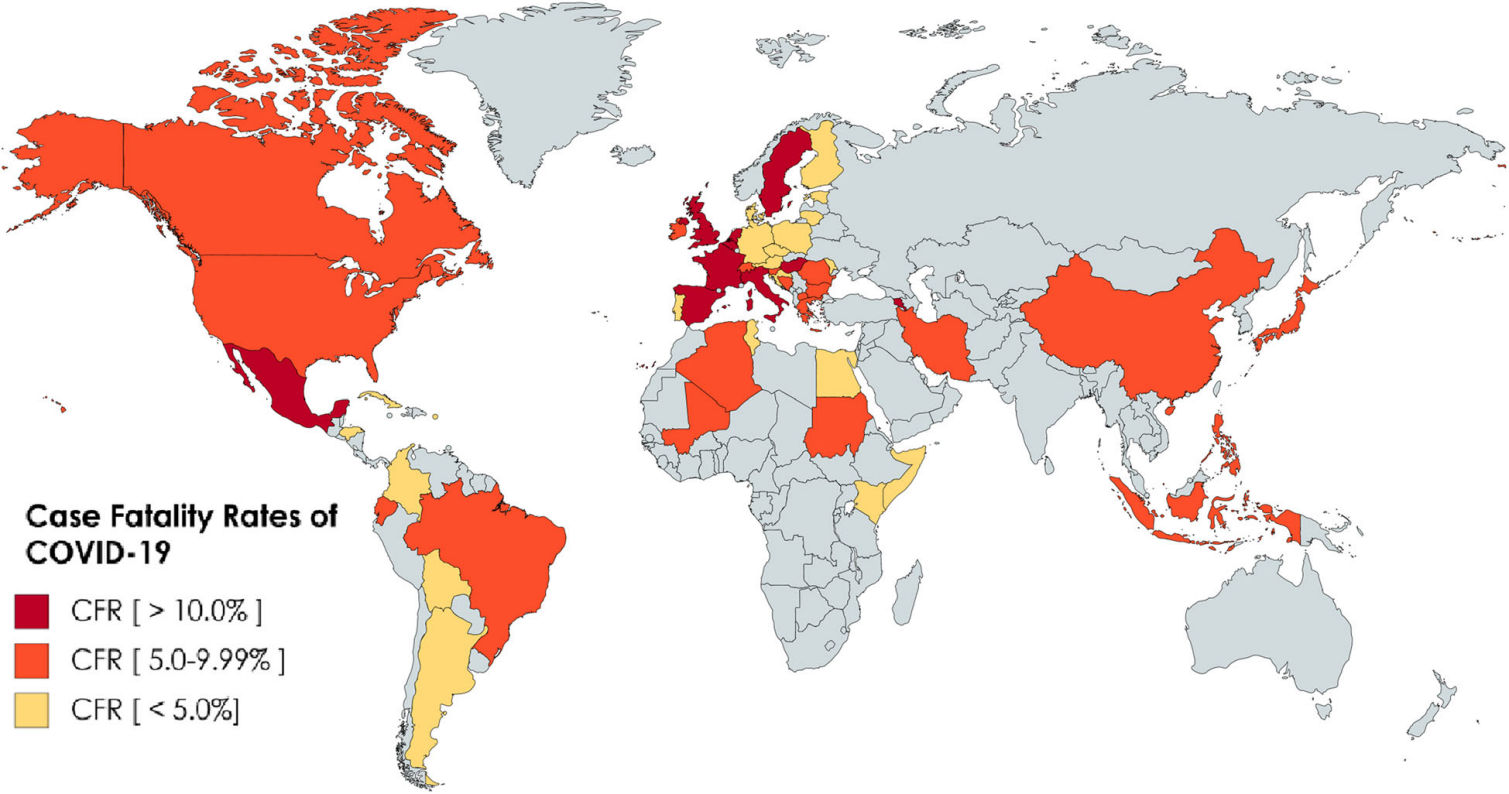
Case Fatality Rate world map, based on data accessed on June 3, 2020. CFR is defined as the number of deaths divided by the number of incident patients within a specified time. CFR is least affected by reporting bias but maybe underestimated by time lag bias due to diagnosing and reporting cases, and the assumption that all cases have been reported, while it may be overestimated by the definition of a case, either confirmed or closed. This map depicts the CFR so far. The highest CFR (> 10%) is reported in Belgium (16.21%), France (15.47%), Italy (14.35%), Hungary (13.54%), the Netherlands, Sweden (11.64%), Spain (11.32%), and Mexico (10.58%). Comparing this map to the IDA prevalence Map in Fig. 1, a stark contrast is seen. Countries with high IDA prevalence have yet to report CFR > 3%. This includes the majority of SSA, excluding Kenya and Mali, as well the South East Asia. Meanwhile, countries with mild anemia prevalence appear to be reporting much higher CFR. There appears to be a connection between severe anemia and lesser CFR, bolstering the notion that the anemic defense is an evolutionary response to acute infection in the endemic setting, a chronic anemia “herd immunity” of sorts that may protect populations rather than a case by case basis

In Figs. 1 and 2, we have mapped out the world wide occurrence of IDA in women of reproductive age (WRA), which is considered all women of child bearing age between the ages of 15 and 49 years (menarche till menopause) (data retrieved from WHO survey), as well as case fatality rate (CFR) of COVID-19 infection (from data retrieved on June 2, 2020) worldwide. Stark and interesting contrasts can be noticed immediately. The great majority of Sub-Saharan Africa (SSA), which consist of all African countries geographically south of the Sahara desert, reports a CFR of < 5%, while having the highest known prevalence of IDA. Interesting juxtapositions are seen in neighboring countries, for example, Papua New Guinea IDA prevalence > 40% versus Indonesia CFR > 5%, the major affection of South America with a CFR > 5% except for Peru and Guyana, which have a severe IDA prevalence of > 40%, and the highest concentration of countries with CFR > 10% in European countries with generally mild IDA prevalence, and this contrast is also seen in America and Canada. These observations suggest a potential connection between IDA prevalence and CFR that needs more evidence to prove. Interestingly, countries with the highest prevalence of IDA seem to be more protected, thus implying that IDA works through a sort of “herd immunity” to protect endemic areas from acute infection, which may also explain why there are lower infection and mortality rates reported in areas like that of SSA.

A systematic analysis of national surveys regarding IDA prevalence among WRA revealed that African countries had the highest prevalence of IDA, including Côte d’Ivoire (49.9%) and Sierra Leone (44.8%) [41]. Of concern is the health care system status and the economic factors in poorer countries of SSA; it is unclear should a connection between COVID-19 and IDA protection exist, how robust this protection would be in the face of under developed healthcare systems and economies. There is evidence that suggests some form of economic and health care instability in SSA that occurred in the aftermath of the Ebola outbreak [42, 43]. Additionally, the African continent appears to be struggling with limited testing capacity. The COVID-19 gold standard test is the nucleic acid-based real-time quantitative polymerase chain reaction (PCR), which is expensive and requires expertise. Even the alternative serological tests with low sensitivity are more available in high income countries. African countries also suffer from low staffing, and inadequate referral systems, which may explain the lower numbers of reported cases. One alternative explanation is that previous pandemics may have actually better prepared African countries in the handling of infectious diseases in the form of swift lockdowns, and the setting up of task forces for pandemic response. Additionally, Africa shows low importation risk, with the highest risk coming only out of Egypt, Algeria, and South Africa [44]. Lastly, Africa’s younger population may explain its lower infection rates, with the median age of Africa being 19.4 years, as compared to 38 and 40 in the USA and Europe, respectively [44, 45].

**Fig. 3:**
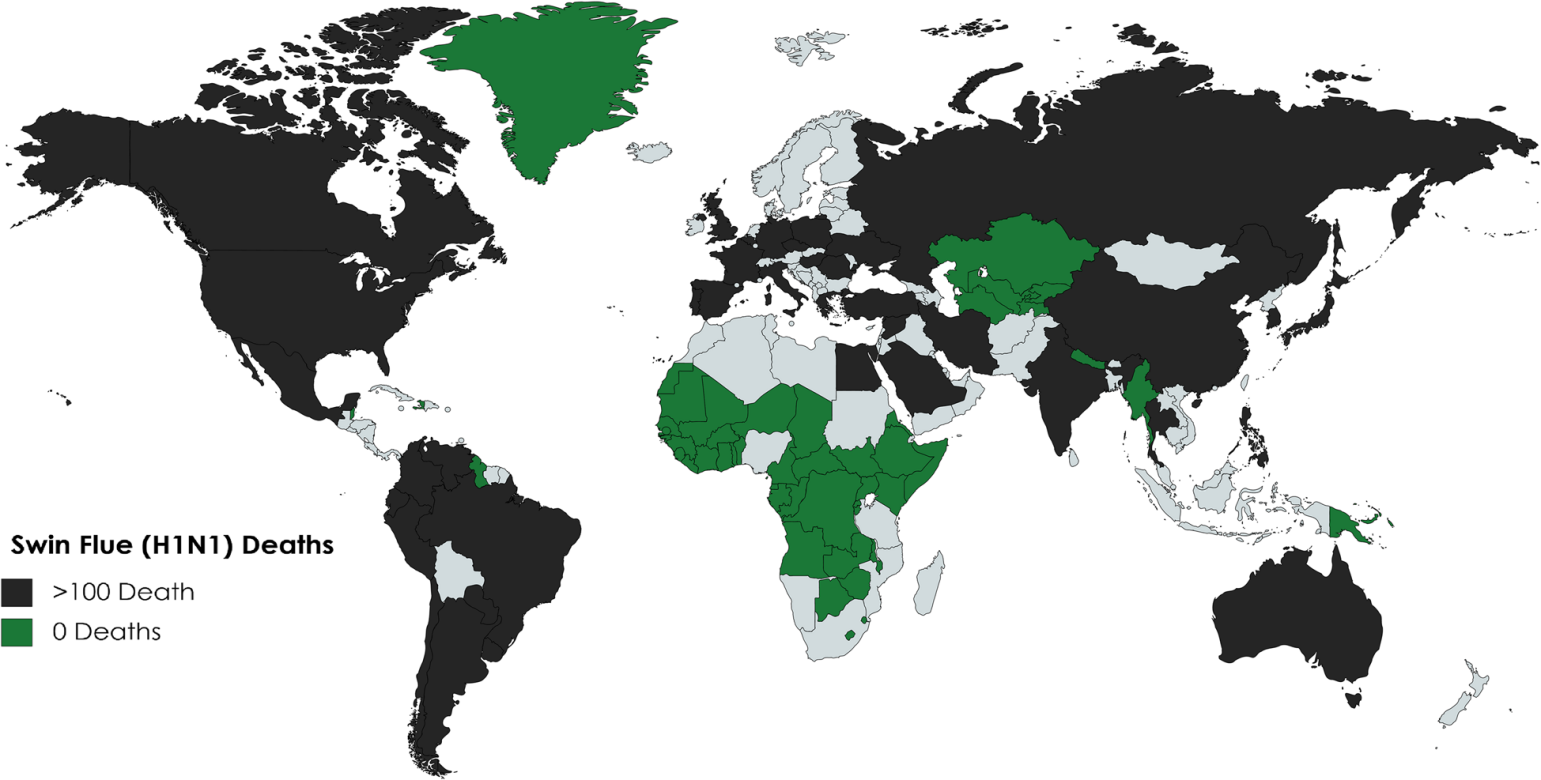
Depicted here are the recorded deaths of the 2009 Swine Flu H1N1 epidemic (source: ECDC). Countries in black suffered more than 100 deaths; deaths were highest in the USA, Brazil, India, and Mexico (> 1000). Countries in green reported no deaths owing to H1N1. Yet again, the highest prevalence IDA countries were very nearly all protected from H1N1 fatalities

**Fig. 4:**
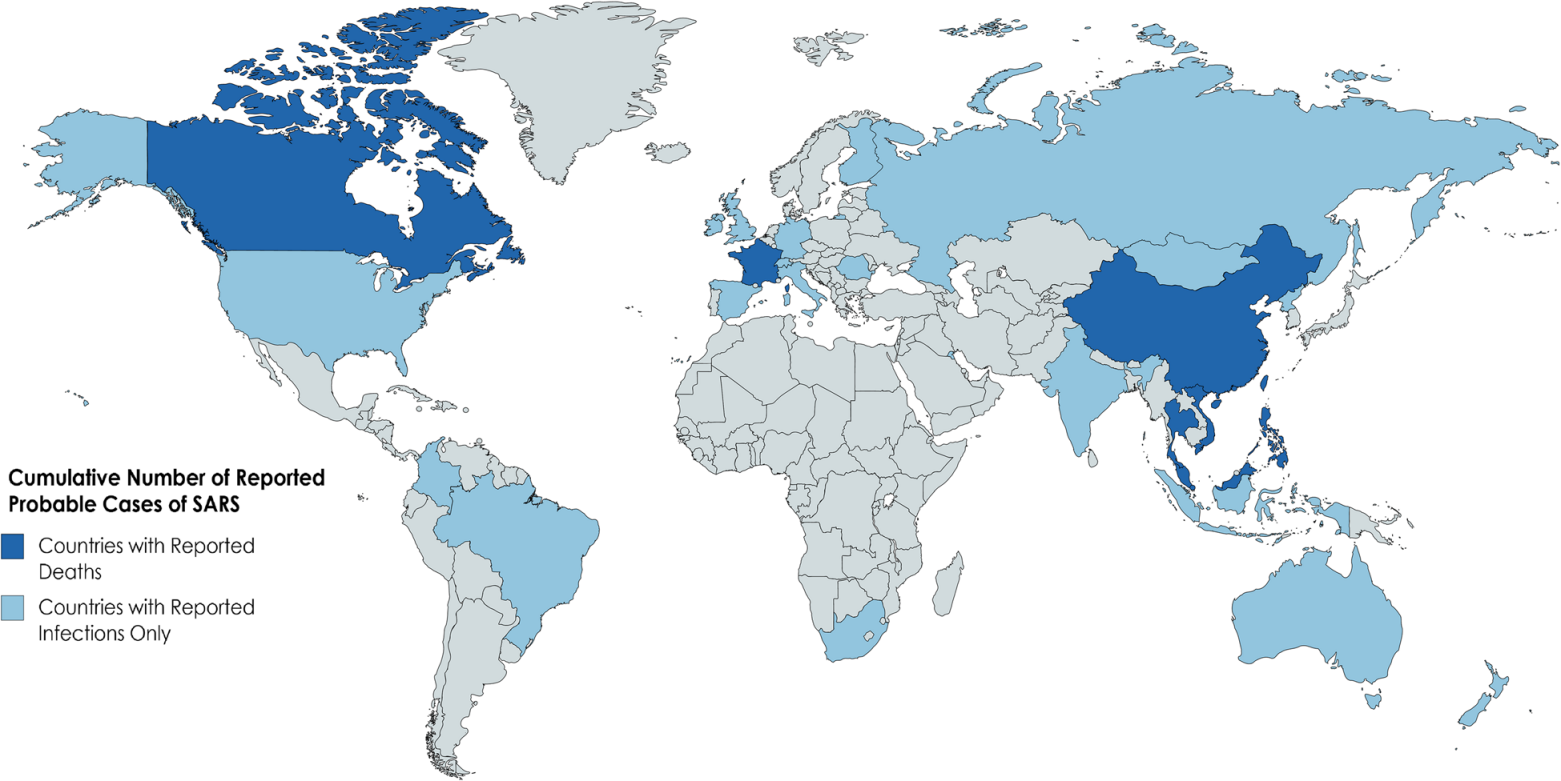
Cumulative number of reported probable cases of SARS based on data from Nov 1, 2002, to July 11, 2003 (source: WHO). Depicted here are countries with reported deaths and infections versus reported infections and no deaths. Countries of high prevalence IDA (see Fig. 1) in SSA were relatively virus free

These observations suggest that it is not the severity of anemia (which COVID-19 patients with severe hypoxemia were observed to have more profound hypoferremia [46]), but rather the severity of the *prevalence* of anemia that is potentially protective, as counties with mild prevalence show a CFR exceeding 5%, as well as the overall distribution given a geographical range. COVID-19 has yet to deeply infect SSA as the geographical range of IDA prevalence is tightly woven in this area. Figures 3 and 4 also depict worldwide affection of the H1N1 virus, and the SARS 2002 epidemic, notably IDA prevalent areas that were minimally affected.

It is suggested that during epidemics with high mortality rates, iron deficiency decreased death and improved survival odds by “affecting the outcome of bacterial superinfection.” This increases for each epidemic faced by a single generation, resulting in an “increased overall fitness of the iron deficient individual” [4]. Additionally, Denic et al. point out that struggles against deadly infections, such as the plague and malaria, may have exerted enough evolutionary pressures to increase the prevalence of an iron deficiency phenotype [4].

### 2. Iron from an evolutionary perspective

From an evolutionary perspective, despite advances in public health to deal with IDA, such as the availability of iron supplements, improved nutrition, and fortification of foods, IDA remains a persistent issue. It is suggested that an iron deficiency phenotype had developed over years of evolution and survived under selection pressures, given the changes to human culture that lead to overcrowding, disease, and agriculture-related iron deficiency diets. It is posited that a failure to adapt to iron deficiency, both genetically and culturally, led to the prevalence of an iron deficiency phenotype.

#### Female menstruation

**Fig. 5:**
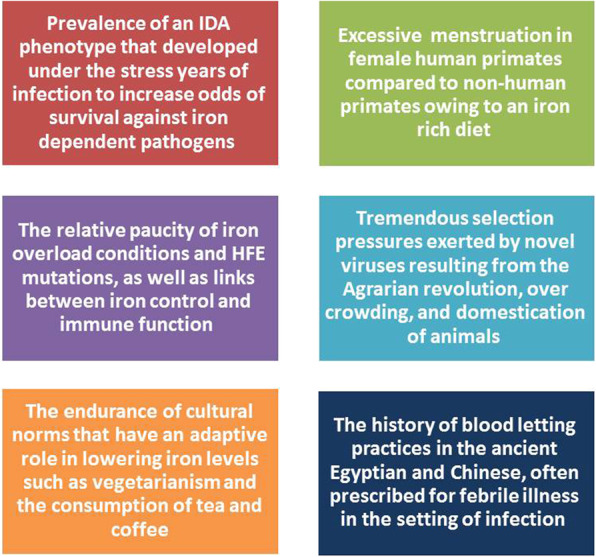
Several evolutionary and social adaptations support the theory that a low iron state may have evolved to mitigate infection. These include the prevalenve of an IDA phenotype that may have protected against iron dependant pathogens, the excessive mentruation oberved in female human primates, the paucity of iron overload conditions, the selection pressures put in place by agriculture and the domestication of animals which would have exposed early humans to novel pathogens, changes in human diet, as well as the persistance of age old blood letting procedures prescribed in ancient medical therapies for the treatment of febrile illness

Denic et al. point towards the excessive menstruation among human females when compared to other primates, such as gorillas and orangutans. Menstruation is the main cause of IDA in females overall. One theory suggests that excessive menstruation in human females was adapted to enhance iron loss. A clear distinction between the herbivore diet of non-human primates and the meat-eating human primate would suggest that a heavier menstruation was adapted to relieve the body of potential excess iron through diet (iron from meat is also more readily available). The origin and purpose of female menstruation is a matter of debate. Evolutionary insights are summarized in Fig. 5.

#### The rarity of human homeostatic iron regulator protein mutations

An additional clue for the evolutionary need for iron deficiency lies in the relative paucity of hemochromatosis genes. Given the essential need for iron in human development, the question becomes why are iron overload diseases less common than iron deficiency phenotypes? The frequency of mutations in the HFE gene worldwide is very low, where in non-European populations the frequency of C28Y and H36D allele mutations is < 1% and 4%, respectively [47]. HFE lies in close proximity to genes involved in immune defense, being located within the major histocompatibility complex (MHC) 1, suggesting a non-immune related role of iron against infection. Hereditary hemochromatosis (HH) was found to affect the immune system as follows: (1) decreased number of Natural Killer T cells (NKT) relating to serum ferritin and transferrin saturation, (2) abnormalities between CD8+ T cell pool, (3) iron-related phenotypic changes in surface expression of molecules on T lymphocytes, (4) lower numbers of circulating and hepatic CD8 cells, and (5) diminished cytotoxic activities of Cytotoxic T lymphocytes (CTL) [48–50].

HFE immunologic role further bolsters the theory of the connection between iron control and the adaptive immune response to pathogens, and implies a regulatory connection between iron metabolism and the immune system. Furthermore, some viruses such as the CMV directly induce degradation of HFE genes to promote iron overload states on a cellular level, providing ample iron needed as its resource [51, 52]. From an evolutionary standpoint, not only has an iron deficiency phenotype prevailed, but also mutations that would increase iron overload states are rare, and have detrimental effects on the immune system.

#### Other genetic considerations

One theory suggests that the presence of pathogens, especially those with high mortality, trigger natural selection in humans to increase resistance alleles, and decrease susceptibility alleles. This may explain the prevalence of anemic conditions throughout geographical or ethnic regions that are targeted by iron-acquiring pathogens. In their paper “The legacy of past pandemics: common human mutations that protect against infectious disease,” Pittman et al. stated that epidemiology of resistant populations and geographical patterns prove that human genetic variations can alter “infection susceptibility and outcomes” [53]. Additionally, sequencing has shown that pathogen-driven selection targets specific loci to increase the alleles for disease, which lends credence to the hypothesis that the maintenance of disease is owed to past pathogen-selective pressures. Background demography is the strongest factor influencing these variations [54, 55].

One clue that supports this comes from a recent finding that respiratory infections trigger hepcidin-meditated blockade of iron absorption in Africans. Prentice et al. discovered that in African children, a hepcidin-dependent physiological block of iron absorption was occurring to the effect of reducing their risk of respiratory infection [56]. An unusually large bolus of iron would be needed to overcome this block, though not without iatrogenic harm [57]. This is another clue to the hypothesis that low iron mitigates infection as a result of infection-related selective pressures. In a recent study, patients with sickle cell disease were found to have low morbidity and mortality with COVID-19. Though these patients were perceived to be high risk, they were “somehow protected from severe symptoms and complications of COVID-19 infection” [58]. One hundred percent of their cohort was African-American, and had anemia with a mean hemoglobin of 7.8 g/dL. It was unclear whether hemoglobin (Hb) F, HbS, or medications were providing a protective effect. Iron deficiency is a common finding in patients with sickle cell disease [59], mostly due to intravascular hemolysis causing urinary loss of iron [60]. Perhaps their low iron status protected against the detrimental hyperinflammatory response to COVID-19 infection, as suggested in this hypothesis.

Other examples include hypoferremia and malaria. The malaria parasite is highly dependent on a small pool of labile iron in the cytoplasm, and is susceptible to nutritional influences that affect this compartment [57].

Among global populations, there are known differences between genetic variations involving iron imbalance, though there is a call for further investigation of these genetic influences among Sub-Saharan Africans due to a significant lack of data [61].

There are other examples of genetic variants developed to protect against past pandemics, which influence infectious diseases today. The best described example is the CCR5-Δ32 allele, believed to have developed 700 years ago in response to Yersinia pestis, the causative organism of the bubonic plague. Individuals that are homozygous for this allele are totally resistant to HIV [62, 63]. The presence of resistance alleles confers some evolutionary advantages. The question remains why these alleles are not fixed. This may be explained by the heterozygous advantage which balances the selection; a notable example being the sickle cell trait which lacks sickle cell disease while protected from malaria. Another reason is simply that the selective pressures fluctuate as pandemics end and new pathogens are introduced, where the previous genetic variant may not be advantageous, or even disadvantageous. For example, while CCR5-Δ32 allele confers immunity to HIV, on the other hand, it increases the risk for West Nile virus infection [64]. Additionally, several generations are needed for the fixation of resistance alleles.

This may explain the contrast between Black Africans who, according this hypothesis, are perhaps protected against COVID-19, as compared to African-Americans who are currently among the high risk groups for COVID-19 infection. One study suggests that African-Americans are at a markedly high risk of infection “that is not fully explained by characteristics of the environment and pre-existing conditions in the population”; perhaps, a genetic influence is responsible [65]. Abdelmassih et al. have outlined the use of a single-cell sequencing in COVID-19. A connection was found between genetic and immune cellular mechanism underlying COVID-19. Substantial variation was found in the rate and severity by which it impacts different demographic groups; predilection was found towards African-Americans [66].

Finally, while resistance alleles may protect against infectious disease, they may increase autoimmune and chronic disease. One example involves coding changes on the *APOL1* gene, which is protective against African sleeping sickness caused by *Trypanosoma brucei rhodesiense*. This same coding variant increases the risk of focal segmental glomerulo-sclerosis and non-diabetic end-stage renal disease in African-Americans [67].

#### iv. Iron, RNA viruses, and the RNA world hypothesis

The RNA world hypothesis posits that, in the evolutionary history of life, it is the RNA molecule that is the origin of life on earth. RNA possesses a multitude of characteristics that befit this theory, such as, its ability to store, transmit, and self-replicate genetic information and its ability to catalyze simple reactions including peptide bonds to form protein [68, 69]. Evidence to this theory further lies in the presence of viroids which are extremely small (246–467 nucelobases), circular, single-stranded, non-coding RNA plant pathogens [70]. Diener proposed that these viroids represent relics of the RNA world [71]. Recently, Forterre theorized, in his “three viruses; three domains” hypothesis, that the last universal common ancestor is an RNA virus [72]. Carrasco-Hernandez et al. recently suggested that RNA viruses are the next likely candidates for upcoming global epidemics. Modern medical technology appears to be particularly challenged by RNA viruses, owing to their rapid adaptive rates and biologic diversity [73]. Studies have identified RNA viruses as the primary cause of all emerging infectious diseases, with a rate of 2–3 novel viruses being discovered yearly [31]. The RNA viral genome has been shown to adapt effectively under selective pressures, owing to their high mutation rates [74]. Many RNA viruses also lack proofreading ability, apparently benefiting from mutability. Coronaviruses are uniquely equipped with a proofreading ability, shown in their Nsp14 protein which functions as a 3′ 5′ exoribonuclease [73]. Mutations within the RNA viral genome can be of great risk to the survival of any virus. Owing to the high fidelity to their code, a single-point mutation can greatly decrease the efficiency of the replication process. In simple terms, each mutation may increase the number of either unviable or viable virions. If the number of unviable virions exceeds the viable ones, the “point of fidelity of equilibrium” tips. Now, loyalty to its genetic code will be to the detriment of the fitness and survival of the entire species, leading to the so called mutational meltdown [73]. Given the unusually long genome of the coronaviruses, and the understanding that the longer the genome the greater the risk of mutation accumulations per replication, it is clear that the presence of the proofreader is of paramount significance to the survival of this species [75]. It is suggested that coronaviruses selectively turn on or off their proofreading ability, allowing them to rapidly adapt to evolutionary stresses effectively, acquiring the right mutations at the right time to ensure adaptability without loss of fidelity to their code and loss of fitness for survival [76].

In his paper titled “Regulation by Iron; RNA Rules the Rust,” Kadner mentions that iron is both a challenge to obtain and maintain, as well as the concern involving production of dangerous reactive oxygen species [77]. Therefore, pathogens must obtain iron given its necessity for their survival, as well as find ways to protect themselves from its adverse effects. RNA appears to affect the expression of genes involved in iron metabolism, as was discovered in the sRNA of *V. cholera* and *E. coli* [78, 79]. Fur proteins are found in many microorganisms, such as *E. coli*, *C. diphtheria*, and *P. aeruginosa*, which have a role in iron-dependent repression. There are two possible settings: (1) in the setting of limited iron, the fur protein is inactive as a repressor and sRNA RhyB is depressed (which is responsible for increasing the turnover of iron-containing proteins that also protect the pathogen from iron damage, such as superoxide dismutase), resulting in increased affinity of iron uptake, and (2) in the setting of excess iron, Fe- Fur binding causes increased expression of iron-containing proteins as well as storage proteins. Furthermore, Fur-binding sites overlap promoter sites for RNA polymerase. These findings suggest the absolute need of iron by RNA and RNA pathogens. RNA pathogens have evolved the perfect mechanisms in dealing with iron-dependent survival challenges [77]. Coronavirus replication was found to be suboptimal in iron-depleted cells. Aconitase is an iron-dependent coronavirus replication protein, whose function was blocked by the use of iron chelators. These findings suggest the iron dependency of coronaviruses [80–82].

### 3. The overarching connection of iron to COVID-19

#### i. Hyperferritinemia syndrome and COVID-19

Ferritin, a universal iron-binding molecule, is responsible for the storage of iron in a biologically active form [83, 84]. Hyperferritinemia syndrome (HFS) is a spectrum of disorders characterized by excessive levels of serum ferritin, which includes macrophage activation syndrome (MAS), adult Still’s disease, septic shock, and multiple organ dysfunction (MOD) [84]. The etiology of HFS depends on the degree of elevation of serum ferritin (mild/moderate/or severe). Severe elevation, seen in HFS, is mainly triggered by viral infection [85, 86]. Similarities between COVID-19 and HFS have recently been noticed. Gomez-Pastora et al. stated that there was elevated serum ferritin (1.5–3.5 times higher) found in all severely-ill COVID-19 patients on admission, and when comparing the ferritin levels of survivors vs. non-survivors, the latter had levels of serum ferritin 3–4 times higher [87]. Ruscitti et al. mentions that COVID-19 shares similar pathogenic mechanisms, clinical picture, and outcomes as HFS, also maintaining ferritin levels in both pathologies has been associated with reduced mortality [2]. The similarities between HFS and COVID-19 have been outlined in Tables 1 and 2 [88]. Although high serum ferritin levels can occur in different types of pathologies, making it a non-specific marker, it should be noted that the degree of elevated serum ferritin tends to be higher when more etiologies of HFS are present [85]. This raises the question, could elevated ferritin levels indicate the prognostic outcome and point out the possible development of complications seen in COVID-19? [84, 88–90] Given the resulting increase in iron availability of these conditions, iron control as suggested may prove to be beneficial and protective [2, 85, 87, 88]. Carcillo et al. demonstrated that correction of ferritin levels in HFS patients resolved their symptoms, whereas uncorrected levels resulted in the development of serious infections [83]. Kernan et al. states that oral iron supplementation during an infection escalates the chances of mortality, while elevated serum ferritin is associated with increased viral/pathogen load, which is also demonstrated in severe COVID-19 patients [83, 88]. It is worthy to note that hyperferritinemia is also implicated in the failure to produce antibodies post vaccination [91] (as seen in studies involving H1N1 vaccine).

**Table 1:** Clinical presentation of COVID-19 and hyperferritinemia-associated syndromes

Clinical presentation	COVID-19 mild–severe	Hyperferritinemia syndromes			
		MAS	MOD	Still’s disease	Septic shock
Fever	+	+	+	+	+
Fatigue	+	+	+	+	+
Headache	+	+	+	+	+
Rash	+	+	+	+	−
Altered mental status	+	+	+	−	+
Dyspnea	+	+	+	−	+
Sore throat	+	−	−	+	−
Joint pain/swelling	+	−	+	−	−

(+) Present and (−) not present (MAS) macrophage activation syndrome (MOD) multi-organ dysfunction

**Table 2:** Laboratory and clinical abnormalities in COVID-19 and hyperferritinemia-associated syndromes [72]

Laboratory/clinical evaluation	COVID-19 severe	Hyperferritinemia syndromes			
		MAS	MOD	Still’s disease	Septic shock
Hyperferritinemia	+	+	+	+	+
Ferritin range (ng/ml)	+ (300–5000)	+ (> 10000)	+ (300–5000)	+ (> 5000)	+ (300–5000)
Hypercytokemia	+	+	+	+	+
Infection triggered	+	+	+	+	+
Multi-organ involvement	+	+	+	+	+
ARDS	+	+	+	+	+
Low/absent NK activity	+	+	+	+	+
ESR/CRP (high/low)	+	+	+	+	+
Abnormal liver function	+	+	+	+	+

(+) Present and (−) not present (MAS) macrophage activation syndrome (MOD) multi-organ dysfunction

This data suggests that COVID-19 may be considered a part of HFS, and its therapy may prove beneficial in the setting of COVID-19. This obervation is an additional proof that optimal iron control may be protective against COVID-19.

#### ii. The link to hepcidin

Hepcidin is the key regulator for iron metabolism. Hepcidin binds to ferroportin, which is the iron export channel found on tissues that prevents the release of iron. Iron would fail to enter the circulation and would remain sequestered inside the cells [92, 93]. In the absence of hepcidin, ferroportin efflux of iron is uninhibited, causing release of iron into the circulation. Therefore, hepcidin determines iron availability and distribution.

As was previously mentioned, pathogens have adapted methods to acquire iron from their hosts. It is even advantageous for some to make use of already present iron-related channels and receptors to acquire this iron, including transferrin and lactoferrin receptors. A distant sequence similarity has been found by Ehsani between the SARS-Cov-2 spike protein and puffer fish hepcidin [3]. This similarity was found at the cytoplasmic tail of the spike protein. Other similarities include cysteine-rich areas on the hepcidin and spike protein, use of furin for activation, the overarching connection between IL-6 and hepcidin and COVID-19 symptoms, and similarities between COVID-19 and altitude illness-related hypoxia, the result of elevated hepcidin. It is suggested that SARS-Cov-2 is utilizing its hepcidin-like similarity to bind to ferroportin receptors on cells, and this remains to be proven. If this is the case, SARS-Cov-2 would be strategically limiting the release of iron from the cells it infects [11]. Cavezzi et al. posit that it is this hepcidin mimicking action that could be the basis for the observed silent hypoxia seen later in severe COVID-19 patients [93]. Cavezzi also notes that the elevated hepcidin in diabetic and obese patients may be a reason for their increased risk of poor outcomes in the setting of COVID-19. Banchini et al. also found that there is an overexpression of hepcidin as well as iron overload in COVID-19 patients [94]. Overexpression of hepcidin in obese, elderly, and diabetic population is a correlating factor that may explain the increased disease severity in these patient groups. Hepcidin is further suggested as a prognostic biomarker in these patients [95].

### 4. Medicine-based adjuvant therapeutic considerations in the setting of infection

#### i. Iron control

Iron chelators are very advanced medications used to prevent the accumulation of excess iron and its toxic effects, and their use has been suggested in the setting of COVID-19 [96, 97]. These drugs decrease iron availability for pathogens, resulting in their inactivation and death [98]. There are many pathogens that are inhibited by iron chelators, such as HIV, Ebola, Herpes simplex virus, TB, *H. pylori*, and HCV [99–101]. Examples of iron chelators include bipyridyl and desferoxamine, which are currently under clinical trial for COVID-19 patients (NCT04333550) [98, 100]. Banchini et al. suggests iron control by exploring both endogenous (insulin, heparin, and erythropoietin) and exogenous options (vitamins D and C, toclizumab, carvedilol), relating to hepcidin control in the setting of COVID-19 [102]. Abbas et al. also suggest the use of iron chelators to lower disease severity in the setting of COVID-19 infection.

Lactoferrin is an iron-binding iron-chelating substance which plays a vital role in the host defense mechanisms [103, 104]. Lactoferrin is known to inhibit pseudotyped SARS-COV with an inhibitory concentration of 50%. The use of lactoferrin was tested in vitro on SARS-Cov-2, which showed inhibition of viral entry via binding to the host cell surface. There is polarizing views regarding the use of iron chelators as adjuvant therapy; chelation should not be employed until there is empirical evidence of increased and relevant levels of iron. A call for anti-hepcidin therapy may be preferred, until actual iron dependency of the RNA coronaviruses is established [105]. There are still many questions that remain to be answered in trials with iron chelators including dosages, timing, and which iron chelator to use [106].

#### ii. Insights from hyperferritinemia

As mentioned before, elevated levels of serum ferritin go hand in hand with iron, so the need to maintain a moderate amount of iron in the body is essential in the therapy of hyperferritinemia, and insights from its therapy may be considered in the treatment of COVID-19 [85, 107]. Remedy used in hyperferritinemia patients is therapeutic plasma exchange (TPE) [108]. Benefits of TPE include reversing disseminated intravascular coagulation (DIC), and the removal of excess amounts of ferritin and free hemoglobin [108]. The 2010 American Society for Apheresis (ASFA) outlines the use of TPE for all hyperferritinemia syndromes [109, 110]. TPE may prove beneficial in the setting of COVID-19 as evidenced by COVID-19 patients who recovered and were discharged after undergoing TPE therapy [101]. Iron chelators may play an additional role here as well, by decreasing the degradation of ferritin by lysosomes, decreasing the production of free radicals, and promoting the downregulation of hepcidin [82].

#### iii. Phlebotomies and erythropoiesis

Phlebotomy is a blood extraction procedure which decreases iron load in patients with excessive iron/ferritin blood levels [111]. With each phlebotomy, ferritin levels decrease by 30–50 points. Tanaka et al. confirms that the use of “petit phlebotomy” is beneficial in Hepatitis-C virus infected patients as it decreases the iron overload (which damages the liver and destroys its protective enzymes) [111, 112]. Phlebotomy decreases total RBC count; as a result, it activates physiological erythropoiesis, which scavenges the body for iron stores resulting in decreased iron availability for pathogens [113]. Iron depletion by phlebotomy has also been shown to reduce insulin resistance and hyperferritinemia [114]. Hadadi et al. concluded that the use of erythropoietin stimulating drugs could attenuate SARS-COV-2 via cytokine modulation, anti-apoptotic effects, and iron redistribution away from the virus [115]. The use of recombinant erythropoietin was shown to have unexplained rapid relief and viral load regression in an 80-year-old male COVID-19 patient with severe anemia. Iron-related abnormalities in patient indices suggest that iron is being depleted on an extracellular level while overloaded on an intracellular level. This is explained by the decrease in hemoglobin and significantly increased index values of serum ferritin [116].

## Conclusion

The insight provided by evolutionary and genetic perspectives, points towards an iron deficiency phenotype that has prevailed throughout generations, providing protection against acute infection. By referring to previous pandemics, a connection can be seen in the general inability of some viruses to invade deeply into high prevalence IDA territories. Human biology seems to have also evolved with this goal in mind, along with cultural and sociological patterns that have upheld the iron deficient phenotype.

Humans may have adapted ways to favor iron loss, while pathogens have adapted methods to effectively acquire iron from their hosts. Coronaviruses are RNA viruses that are highly dependent on iron. Evidence that point to an overarching role of iron in COVID-19 infection includes hyperferritinemia in severe cases, similarities between COVID-19 and hyperferritinemia syndromes, and hepcidin overexpression that may also be responsible for the increased risk of disease in diabetic, obese, and elderly patients. Given this understanding, targeting iron from a therapeutic stand point may prove decisive as part of adjuvant therapy against such viruses; however, more robust evidence is needed. These would include the use of iron chelators, or therapy which would sequester iron away from pathogens like erythropoietin, and targeting hepcidin control through several endogenous and exogenous means via anti-hepcidin agents. The observation of elevated ferritin warrants their use as prognostic markers for disease progression in COVID-19 patients. More robust research is needed to link the potential of iron control and the therapy of COVID-19 patients.

Overall, low iron is theorized to mitigate infection. It should be noted that this hypothesis still requires more evidence. Namely, iron itself is necessary for immune cell proliferation, and counter evidence suggests that low iron increases the risk of some infection. What remains to be elucidated are as follows: which low iron state is conferring the protective effect?, is it overt iron deficiency anemia or covert iron deficiency without anemia? And which pathogens are affected by this low iron state? These are the questions that remain to be answered by this hypothesis. This theory ultimately suggests that evolutionary stressors have promoted genetic variations in the form of resistant alleles. If low iron mitigates infection, then the areas with high iron deficiency anemia prevalence may be protected against some types of infection, such as Sub-Saharan Africa. Therefore, to enhance this theory, further investigations are needed to determine the genetic variants responsible for iron imbalance among susceptible populations. Focus on iron indices in COVID-19 patients is also necessary to provide a clear picture of the iron metabolism of these patients before the suggestion of iron control in the clinical setting.

## Data Availability

Not required

## Abbreviations

ARDS: Acute respiratory distress syndrome
CMV: Cytomegalovirus
COVID-19: Coronavirus Disease 2019
DIC: Disseminated intravascular coagulation
HFE: High Fe2+
HFS: Hyperferritinemia syndrome
HH: Hereditary hemochromatosis
HIV: Human immunodeficiency virus
IDA: Iron deficiency anemia
PCR: Polymerase chain reaction
RNA: Ribonucleic acid
SARS-COV-2: Severe Acute Respiratory Syndrome Coronavirus 2
SSA: Sub-Saharan Africa
TPE: Therapeutic plasma exchange
WRA: Women of reproductive age
